# Population-Based Data Reveal Factors Associated with Organised and Non-Organised Colorectal Cancer Screening: An Important Step towards Improving Coverage

**DOI:** 10.3390/ijerph18168373

**Published:** 2021-08-07

**Authors:** Thuy Ngan Tran, Guido Van Hal, Marc Peeters, Svetlana Jidkova, Harlinde De Schutter, Sarah Hoeck

**Affiliations:** 1 Family Medicine and Population Health (FAMPOP), Faculty of Medicine and Health Sciences, University of Antwerp, 2610 Antwerp, Belgium; guido.vanhal@uantwerpen.be (G.V.H.); sarah.hoeck@uantwerpen.be (S.H.); 2 Centre for Cancer Detection, 8000 Bruges, Belgium; Svetlana.Jidkova@UGent.be; 3 Department of Oncology, Antwerp University Hospital, 2650 Antwerp, Belgium; marc.peeters@uza.be; 4 Integrated Personalized & Precision Oncology Network (IPPON), University of Antwerp, 2610 Antwerp, Belgium; 5 Department of Public Health and Primary Care, Ghent University, 9000 Ghent, Belgium; 6 Research Department, Belgian Cancer Registry, 1210 Brussels, Belgium; Harlinde.DeSchutter@kankerregister.org

**Keywords:** colorectal cancer, cancer screening, organised screening, non-organised screening, faecal occult blood test, screening coverage, cancer health disparities

## Abstract

We investigated factors associated with organised and non-organised colorectal cancer screening using faecal occult blood tests, based on data from 308 municipalities in Flanders (6.6 million residents, 57% of Belgium) during 2015–2017. Logistic regression with generalized estimating equations was used to assess the associations between municipal characteristics and organised and non-organised screening coverages. Factors associated ***negatively with both organised and non-organised screening***: percentage of people aged 70–74 in the target population [OR (odds ratios) = 0.98, 95%CI (confidence interval): 0.97–0.99 and OR = 0.98, 95%CI: 0.96–0.999, respectively]; ***negatively with organised screening***: average income [OR = 0.97, 95%CI: 0.96–0.98], percentage of people with a non-Belgian/Dutch nationality [OR = 0.962, 95%CI: 0.957–0.967]; ***positively with organised screening***: percentages of men in the target population [OR = 1.13, 95%CI: 1.11–1.14], jobseekers [OR = 1.12, 95%CI: 1.09–1.15] and people with at least one general practitioner (GP) visit in the last year [OR = 1.04, 95%CI: 1.03–1.05]; ***positively with non-organised screening***: number of patients per GP [OR = 1.021, 95%CI: 1.016–1.026], percentage of people with a global medical dossier handled by a preferred GP [OR = 1.025, 95%CI: 1.018–1.031]. This study helps to identify the hard-to-reach subpopulations in CRC screening, and highlights the important role of GPs in the process of promoting screening among non-participants and encouraging non-organised participants to switch to organised screening.

## 1. Introduction

Worldwide, colorectal cancer (CRC) ranks third in terms of cancer incidence and second in terms of mortality [1]. In Flanders, colorectal cancer was the second most common cancer in females and third in males in 2018, with low incidences before the age of 50 (<22.4/100,000 person-years (py) for ages 45–49) but gradually increasing rates for older age groups. Incidence rates ranged, for males and females respectively, from 59.9/100,000 py and 48.0/100,000 py for ages 50–54 up till 280.5/100,000 py and 184.8/100,000 py for ages 70–74 [2]. 

Flanders, the most populated region of Belgium (57% of the country’s population) [3], had 4954 new CRC cases and 1617 CRC deaths in 2017 [2]. Regular screening is an excellent preventive intervention for CRC: the 5-year relative survival rate for stage I CRC is 94.7% while for stage IV CRC it is only 16.2% (Flanders, 2000–2018) [2]. Organised screening is the only screening strategy for CRC recommended by the European Council since it ensures equity of access and quality control [4,5]. In Flanders, the organised CRC screening programme has been in place since 2013, offering a free biennial faecal occult blood test (FOBT, immunochemical type) to all eligible individuals aged 50–74.

Despite the recognised benefits of organised CRC screening, only just over half of the target population in Flanders participate in the organised screening programme [6]. Some of them, instead, undergo a non-organised FOBT. The main issues with non-organised FOBTs are that they are not free-of-charge; results and follow-up information are not systematically registered, and quality is not systematically controlled by the organised CRC screening programme, the cancer registry or any other authorities. Therefore, it is crucial to identify factors associated with organised and non-organised FOBT screening. Unfortunately, comprehensive data on non-organised FOBTs are currently lacking [7].

A unique strength of the CRC screening programme in Flanders is the ability to obtain data on non-organised FOBTs (prescribed by GPs and specialists). In this study, we investigated factors associated with organised and non-organised FOBT screening coverages at a municipality level. Our findings will help to guide targeted interventions to increase CRC screening among non-participatory individuals or encourage non-organised participants to switch to organised screening.

## 2. Methods

### 2.1. Flanders and Its Organised CRC Screening Programme

Flanders is the most populated region of Belgium (6.6 million, 57% of Belgian population) [3]. It comprises 308 municipalities with populations varying from ~90 to 520,900, of which 19–40% were at eligible ages for CRC screening (2015–2017). The organised CRC screening programme in Flanders has been in place since 2013 and is coordinated by the Centre for Cancer Detection. The programme offers a free FOBT (immunochemical type) every two years to all citizens aged 50–74 using a centralized invitation procedure (target ages were extended gradually from 56–74 in 2013 to 50–74 in 2020). During the study period, the target screening ages were 56–74 in 2015–2016 and 55–74 in 2017. People were excluded from the screening invitation list if they had had a stool test in the past two years, a virtual colonoscopy in the past four years or a complete colonoscopy in the past ten years, were diagnosed with CRC in the past ten years or had undergone a colectomy (excluded permanently).

### 2.2. Study Population and Data Sources

We included data from all 308 municipalities in Flanders in 2015–2017. Data on organised FOBT screening coverage, gender and age-specific proportions of the screening population were obtained from the Centre for Cancer Detection.

Data on non-organised FOBTs, identified by nomenclature codes used in health insurance claims, are available at the Belgian Cancer Registry which receives these data from the health insurance companies. In Flanders, individuals who have had an FOBT in the past two years, regardless of whether it was an organised or non-organised test, are excluded from screening invitations. Four times per year, the Centre for Cancer Detection receives data on non-organised FOBTs from the Belgian Cancer Registry in order to prepare the screening invitation list. These data were used in the current study as a source of information regarding non-organised FOBTs.

Data on other demographic, socioeconomic and health-related municipal characteristics were retrieved from the publicly accessible database of the Flemish provincial authorities (https://provincies.incijfers.be/databank (accessed on 17 August 2020)) and were linked to the data on screening coverage.

### 2.3. Main Outcomes

The main outcomes are the annual organised CRC screening coverage and the annual non-organised CRC screening coverage from 2015 to 2017.

### 2.4. Determinants Considered

**Figure 1:**
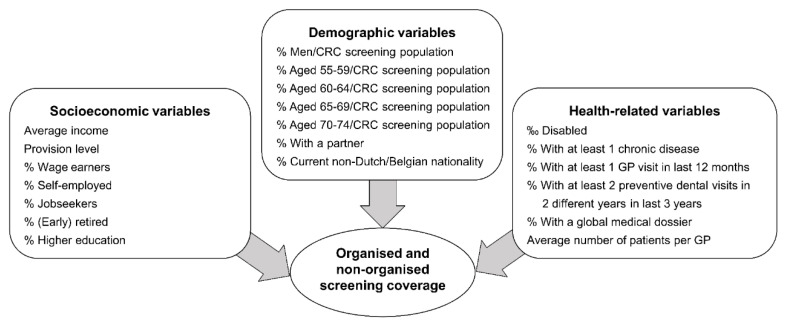
Potential municipal characteristics associated with organised and non-organised colorectal cancer screening using faecal occult blood test.

Figure 1 presents twenty demographic, socioeconomic and health-related municipal characteristics included as potential factors associated with organised and non-organised FOBT screening coverages.

#### Variable Explanation

Proportions of genders and age groups were measured for the target CRC screening population in each municipality. Other variables were measured for the total population of a municipality and were used as a proxy for the characteristics of the target CRC screening population. Current nationality combines Belgian and Dutch because language and cultural barriers seem irrelevant for Dutch people (Dutch is the official language in Flanders) [8]. Municipal average income is calculated by the total net taxable income divided by the number of inhabitants. Municipal provision is measured by the available supply as regards education, care, public and commercial services, personal services, hotels-restaurants-cafes, retail trade, culture/recreation and sport; and is classified into seven levels [9]. Distribution of positions in the labour market was characterized by the percentage of the four main positions (wage-earners, self-employed, jobseekers and (early) retired). The percentage of residents aged 18–24 studying at a college/university (higher education) was used as a proxy for education level. Disabled people are registered by the Directorate General for Disabled Persons as losing at least one third of the average earning capacity or being unable to perform daily activities. GP visits and preventive dental visits were defined as the percentage of people who had had at least one GP visit in the last 12 months and at least three preventive dental visits in two different years in the last three years, respectively. The global medical dossier formally indicates the patient’s preferred GP, who handles the dossier and follows the patient’s medical history. Other variables are self-explanatory (details on https://provincies.incijfers.be/databank (accessed on 17 August 2020)).

### 2.5. Covariates

We used the causal directed acyclic graph (DAG) approach to identify covariates for adjustment when assessing the associations between municipal characteristics and the organised/non-organised screening coverages. We constructed causal diagrams of the study variables and selected covariates, taking into account the between-variable relationships. The final list of covariates for adjustment is presented in Table 1. The detailed DAGs showing the pathways among the variables before and after adjusting for covariates are included in Supplementary Figure S1. The relationships among the included variables were defined based on our prior knowledge about the Flemish context and the organised programme, independently of the study data. The use of the DAG approach helps to avoid bias due to over-adjusting for variables that may behave statistically like confounders (collider bias) [10].

### 2.6. Statistical Analysis

#### 2.6.1. Missing Data

For privacy reasons, figures were not displayed for cells with <5 events. As missing data was minimal (1.5%) and solely due to privacy concerns, complete case analysis was applied.

#### 2.6.2. Sample Size

For logistic regression, at least 10 outcome events per determinant are required [11]. We included 20 determinants while having 308 municipalities that carried data on organised and non-organised screening coverages (study outcomes). Therefore, our sample size could provide sufficient statistical power.

#### 2.6.3. Main Analysis

Continuous variables were described with medians (ranges) and categorical variables were described with numbers (proportions). Each person was assigned a screening status for organised screening (covered versus not covered by an organised FOBT) and for non-organised screening (covered versus not covered by a non-organised FOBT), so the study outcomes are grouped binomial. To evaluate the associations between the determinants and the annual screening coverage of the two screening strategies, we used logistic regression with generalized estimating equations (GEE) to account for the correlation of repeated measurements of municipalities’ characteristics and screening coverage each year during the study period. Crude and adjusted odds ratios (ORs) were reported with 95% confidence intervals (95% CIs). Multicollinearity in multivariate models was checked using variance inflation factors (VIFs). *p*-values less than 0.05 (two-sided) were considered statistically significant. All analyses were performed with R (version 4.0.2 (R Foundation for Statistical Computing, Vienna, Austria)).

### 2.7. Ethics

For secondary aggregated data, ethical approval was not required. Our reporting adheres to the STROBE guidelines for observational studies [12].

## 3. Results

### 3.1. Municipal Characteristics

**Figure 2:**
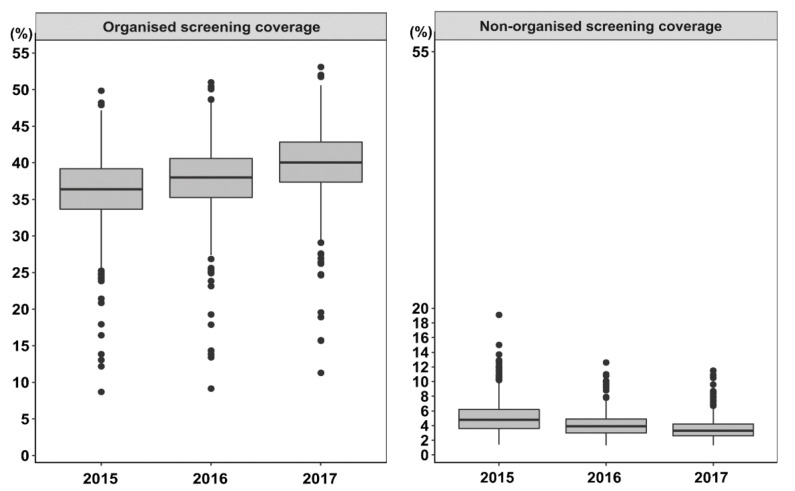
Organised and non-organised faecal occult blood test screening coverage of 308 municipalities in Flanders, 2015–2017. The outlier points show that there are a number of municipalities with extremely low organised screening coverage (below 1.5 times the interquartile range) and municipalities with extremely high non-organised screening coverage (above 1.5 times the interquartile range).

The demographic, socioeconomic and health-related characteristics of the 308 study municipalities in 2015–2017 are summarised in Table 2. Their organised and non-organised FOBT screening coverages are presented in Figure 2. The median organised screening coverage increased from 36.4% in 2015 to 38.0% in 2016 and 40.1% in 2017, whereas the median non-organised screening coverage decreased from 4.8% in 2015 to 3.9% in 2016 and 3.3% in 2017. A wide variation in organised and non-organised screening coverages existed among municipalities. There were municipalities with extremely low organised screening coverage and municipalities with extremely high non-organised screening coverage (presented with outlier points in Figure 2).

### 3.2. Factors Associated with Organised and Non-Organised Screening Coverage

**Figure 3:**
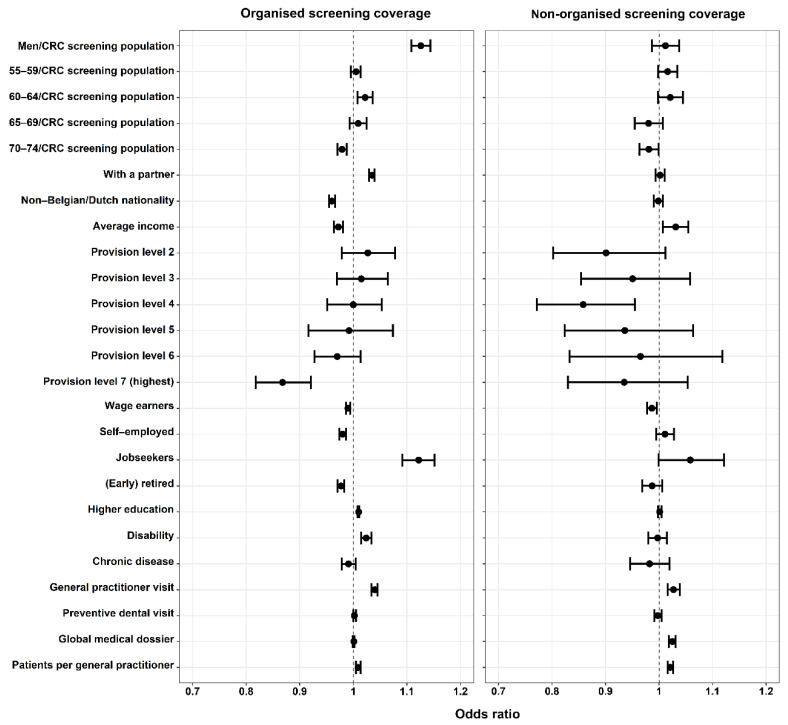
Associations between municipal characteristics with organised and non-organised screening coverages, presented with adjusted odds ratios and 95% confidence intervals.

Multicollinearity in multivariate models was low (VIFs: 1.0–5.2). Associations between municipal characteristics and organised and non-organised FOBT screening coverages are graphically presented in Figure 3 and detailed in Table 3 and Table 4.

**Factors associated with both organised and non-organised screening coverages:**

A higher average income was associated with a lower organised screening coverage (OR = 0.97, 95%CI: 0.96–0.98) but a higher non-organised screening coverage (OR = 1.03, 95%CI: 1.01–1.06). A higher percentage of people aged 70–74 in the target screening population was associated with lower screening coverages by both screening strategies (organised screening: OR = 0.98, 95%CI: 0.97–0.99; non-organised screening: OR = 0.98, 95%CI: 0.96–0.999).

A higher percentage of people with at least one GP visit in the last year was associated with higher screening coverages by both screening strategies (organised screening: OR = 1.04, 95%CI: 1.03–1.05; non-organised screening: OR = 1.03, 95%CI: 1.02–1.04). Compared to organised screening coverage, the association between non-organised screening coverage with average number of patients per GP (OR = 1.021, 95%CI: 1.016–1.026) was more pronounced.

**Factors associated with only organised screening coverage:**

The highest equipment level (OR = 0.87, 95%CI: 0.82–0.92) and a higher percentage of people with non-Belgian/Dutch nationality (OR = 0.962, 95%CI: 0.957–0.967) were associated with a lower organised screening coverage.

Regarding the distribution of labour positions, a higher percentage of jobseekers was associated with a higher organised screening coverage (OR = 1.12, 95%CI: 1.09–1.15). Organised screening coverage was also positively associated with education level (OR = 1.010, 95%CI: 1.008–1.011), the percentage of people with a partner (OR = 1.035, 95%CI: 1.029–1.040), disability (OR = 1.024, 95%CI: 1.015–1.034) and more men in the target CRC screening population (OR = 1.13, 95%CI: 1.11–1.14).

**Factors associated with only non-organised screening coverage:**

A higher percentage of people with a global medical dossier handled by a preferred GP was associated with a higher non-organised screening coverage (OR = 1.025, 95%CI: 1.018–1.031).

## 4. Discussion

Our findings suggest several hard-to-reach subpopulations in CRC screening. Higher average income, lower average education level and a higher percentage of people with non-Belgian/Dutch nationality were associated with a lower organised screening coverage. More older people (70–74) in the target population were associated with lower coverages for both organised and non-organised screening. GPs were shown to have an important role in improving CRC screening coverage: a higher percentage of people with a GP visit in the last year was associated with higher coverage for both screening strategies, whereas a higher average number of patients per GP and a high percentage of people with a global medical dossier handled by a preferred GP were associated with a higher non-organised screening coverage.

In this study, we could not compare the organised and non-organised FOBT screening coverages in Flanders with other regions/countries because they do not have data on non-organised FOBTs and have therefore not reported these indicators. However, in terms of screening uptake, the FOBT screening uptake in Flanders was 51.5–54.6% (2015–2018) [6], within the range of screening uptake reported in other European countries 15.3–71.3% [5].

A lower FOBT screening coverage (both organised and non-organised) was observed in municipalities with more people in the oldest target age group (70–74). The negative association between older age and participation in FOBT screening has also been reported in other European countries [5]. Older people often suffer multiple health issues and have a lower perceived life expectancy, which is linked to poorer CRC screening [13]. Other health priorities might also limit their screening participation. However, it should be noted that the benefits of CRC screening for this group still outweigh its risks. At age 75, a Flemish man and woman still have an average life expectancy of 9.9 and 12.5 years, respectively [14]. The higher CRC incidence in the group aged 70–74 could also contribute to the lower organised screening coverage in municipalities with more people aged 70–74 in the target screening population, since those diagnosed with CRC were excluded from the invitation list of the screening programme and could no longer participate and be counted in the category “coverage by organised screening”.

The success of organised CRC screening programmes in removing financial barriers to screening with the provision of free FOBTs has been proven in previous studies in which no association between organised FOBT uptake and income was found [7,15]. Our study (Flanders, Belgium), in agreement with two other studies (Korea and Manitoba, Canada) [7,16], even found that income was associated negatively with organised but positively with non-organised screening. The increase in non-organised screening coverage with income is to be expected, since non-organised FOBTs are not free-of-charge. However, the fact that this is observed alongside a decrease in organised screening coverage is worrisome. As organised FOBTs are population-based and free-of-charge, some people might perceive these organised FOBTs to be of lesser quality and opt for non-organised tests [17]. Further research is needed to test this hypothesis and if it is proven, it is crucial for the Flemish screening programme to reassure the target population that the quality of the organised tests is systematically reviewed by the screening programme, and highlight the additional advantages of having their screening history, results and follow-up information systematically monitored.

Our study found a lower organised FOBT screening coverage in municipalities with a higher percentage of people with non-Belgian/Dutch nationality. The negative association between non-Belgian/Dutch nationality and organised CRC screening has also been shown in a previous Flemish study at the individual level [8]. Two main reasons for FOBT non-participation reported by migrants in Flanders are language issues and embarrassment when talking about CRC screening and stool samples [18]. As screening invitations are written in Dutch, many non-Dutch speaking people expressed a lack of screening information. Some even mistook the invitations for advertisements and discarded them. Older migrants admitted that they depended on their children to translate screening materials but found it uncomfortable talking about CRC screening and stool collection [18]. Language issues also limit migrants’ communication with GPs and prevent them from obtaining screening information.

A lower organised screening coverage in migrants may also explain the lower organised screening coverage in municipalities with the highest equipment level. These municipalities, with better job opportunities and access to services, have a higher percentage of residents with nationalities other than Belgian/Dutch (9.2%) compared to municipalities with a lower equipment level (2.9–4.8%) [19]. It is also possible that with more accessible and concentrated healthcare services, more people underwent ‘preventive’ colonoscopies and were excluded from organised screening. 

In agreement with previous studies at the individual level [20,21], we found a positive association between education level and FOBT screening coverage. This suggests that the gap in FOBT screening between people with high and low education levels still exists and needs to be addressed. In general, it is easier for highly educated people to obtain and comprehend screening information. They also understand the importance of screening better.

Along with the well-reported association between FOBT screening (both organised and non-organised) and GP visits [15,18], we found pronounced associations between non-organised screening coverage with the average number of patients per GP and the percentage of people who had a global medical dossier handled by a preferred GP. On the one hand, GPs showed a positive impact on promoting CRC screening in the target population. On the other hand, it appeared that despite the availability of the organised programme, some GPs still prescribed a non-organised FOBT to patients instead of referring them to the organised programme. These likely include older GPs who have a large number of patients but are less familiar with screening practices. A previous evaluation also revealed that in Flanders, some GPs were unaware of specific elements of the screening programme. While the recommended follow-up after a positive organised FOBT is a colonoscopy, some GPs prescribed a non-organised FOBT, hoping for a second positive result in order to convince patients to undergo a colonoscopy. Others did not know that in the case of a lost test, GPs or patients can contact the organised programme for another free test [14]. Our findings highlight the importance of providing GPs with sufficient and accurate information about the organised screening programme so that they can effectively assist patients in making informed decisions about screening.

Regarding labour position distribution, municipalities with a higher percentage of jobseekers had a higher organised FOBT screening coverage, while municipalities with a higher percentage of wage earners and the self-employed had a lower organised FOBT screening coverage. One possible reason is that jobseekers have more time to complete a stool test at home. Less time for sample collection at home has been reported as a reason for individuals not choosing FOBT as their preferred CRC screening method compared to (hypothetical) blood and saliva sampling [22]. A previous Flemish study at the individual level also found negative associations between organised FOBT screening with wage earners and being self-employed [8]. It was not possible to compare our findings regarding the association between employment and FOBT screening with other countries due to different systems of employment classification [15,16,20].

The positive association between having a partner and organised FOBT screening has been well-reported in previous studies [20,23]. In this study, we also found a higher organised FOBT screening coverage in municipalities with a higher percentage of people with a partner. Those who have a partner have a higher sense of responsibility towards themselves and their partner and are more likely to engage in healthy lifestyles [24]. Communication between a couple can also promote each other’s awareness and involvement in screening [24]. Co-invitation (inviting partners together) has been suggested as a potential measure to increase CRC screening uptake [25].

Prior literature has reported inconsistent results regarding the association between having a disability and FOBT screening due to different ways of classifying disabilities (type and severity) [26,27,28]. Although we could not classify disabilities further due to data unavailability, we found a general positive association between the percentage of people with a disability and organised screening coverage. People with disabilities normally value health more highly and are more conscious about preventive care. They contact GPs/specialists more frequently and are more likely to receive screening recommendations [21]. Moreover, disabled people may have financial problems and appreciate the free organised FOBT. This test is also convenient for them since it is mailed to their home and no transportation is needed.

An interesting result that we found with the use of data at the municipality level is that more men in the screening population were associated with a higher organised screening coverage. This finding seemed counter-intuitive at first sight, since previous studies have shown that women are more likely to participate in CRC screening than men [8,20,23,29,30,31]. However, a closer data inspection revealed that in Flanders, within a municipality, the screening coverage in women was higher compared to men, but among municipalities, more men in the screening population were associated with a higher screening coverage in both men and women, leading to a higher overall screening coverage. A higher rate of positive screening results and adenoma/CRC detection has been consistently reported in men compared to women [5,6,32]. One possible explanation for our finding is that in municipalities with more men in the screening population, resulting in a higher rate of positive results and adenoma/CRC detection in men, people are more exposed to CRC-related information and experiences, and are therefore made more aware and more likely to participate in screening.

A key strength of this study is the ability to obtain data on non-organised FOBT screening, which is currently lacking in other regions/countries. Moreover, the use of administrative data eliminated selection and recall bias associated with self-reported data. Since the amount of missing data was small (1.5%), selection bias due to missing data was unlikely. Collider bias was avoided with the use of DAG to identify covariates for adjustment (details in Methods).

Several limitations need to be acknowledged. Firstly, our results at the municipality level may be subject to ecological fallacy, meaning some associations may not hold true at the individual level. Secondly, most of the independent variables were measured for the complete municipality population and used as proxies for the screening population. Nevertheless, the surrounding environment has proven to influence individuals’ health behaviours and decisions significantly [33], and our results substantiate previous findings at individual level. Thirdly, we could not include non-organised FOBTs ordered from pharmacies/online because data were unavailable. Non-organised screening coverage might be underestimated. Fourthly, data on reasons for the prescription of non-organised FOBTs was unavailable, so we could not judge whether a non-organised FOBT was taken for a screening or diagnostic/therapeutic reason. Some of the non-organised FOBTs might be appropriately prescribed for a specific indication which fell outside the remit of the organised screening programme. Finally, although it has been well reported that the younger group (50–59) participate less in FOBT screening [5,8,15,29,30], we could not fully assess the association between this age group and FOBT screening coverage since ages 50–54 were not yet included in the target age range in the study period.

## 5. Conclusions

Our findings showed that higher average income, lower education level and non-Belgian/Dutch nationality were related to a lower organised FOBT screening coverage while older age (70–74) was related to lower screening coverages for both organised and non-organised screening. GP visits were positively associated with screening coverages for both screening strategies, highlighting the important role of GPs in promoting CRC screening among the target population. The associations between the average number of patients per GP and having a global medical dossier handled by a preferred GP with non-organised screening coverage were more pronounced compared to organised screening coverage. Efforts are needed to provide GPs with sufficient and accurate information about organised and non-organised CRC screening so that they can effectively assist patients in making informed decisions about screening. It is also crucial to identify and address barriers to CRC screening, especially organised CRC screening, in the subpopulations with lower screening coverage. The aim is to first and foremost promote screening among non-participants so that they are covered by screening (regardless of the screening strategy). Additionally, from both economic and organisational points of view, those who have undertaken non-organised FOBTs for CRC screening should be encouraged to switch to organised screening. Future research at a lower geographical or individual level and more in-depth investigation into the barriers to FOBT screening in specific subpopulations are needed to verify our findings.

## Figures and Tables

**Table 1:** List of covariates for adjustment in multivariable analyses to estimate the association between each municipal characteristic (listed under ‘main determinant of assessment’) and organised/non-organised FOBT screening coverages.

Main Determinant of Assessment	Covariates for Adjustment in Multivariable Analyses
Men/CRC screening population	Year
Age groups/CRC screening population	Provision level, year
With a partner	Age groups/CRC screening population, year
Current non-Belgian/Dutch nationality	Provision level, year
Average income	With a partner, age groups/CRC screening population, current non-Belgian/Dutch nationality, chronic disease, disability, education level, provision level, men/CRC screening population, position in labour market, year
Provision level	Year
Position in labour market	Age groups/CRC screening population, current non-Belgian/Dutch nationality, disability, education level, provision level, men/CRC screening population, year
Education level ^§^	GP visit, with a partner, age groups/CRC screening population, average income, current non-Belgian/Dutch nationality, chronic disease, disability, provision level, men/CRC screening population, global medical dossier, position in labour market, preventive dental visit, year
Disability	Provision level, year
Chronic disease	Age groups/CRC screening population, current non-Belgian/Dutch nationality, disability, education level, provision level, men/CRC screening population, year
GP visit	Age groups/CRC screening population, average income, chronic disease, disability, education level, men/CRC screening population, preventive dental visit, year
Preventive dental visit	With a partner, age groups/CRC screening population, current non-Belgian/Dutch nationality, chronic disease, education level, men/CRC screening population, global medical dossier, position in labour market, year
Global medical dossier	With a partner, age groups/CRC screening population, current non-Belgian/Dutch nationality, chronic disease, education level, men/CRC screening population, position in labour market, preventive dental visit, year
Average number of patients per GP	Provision level, year

^§^ For education level, covariates for adjustment could only be identified for estimating the direct effect (not mediated via other variables) of this factor on the study outcomes.

**Table 2:** Demographic, socioeconomic and health-related characteristics of all 308 municipalities in Flanders, 2015–2017.

	Median (IQR), unless Stated Otherwise		
	2015 (*n* = 303) ^†^	2016 (*n* = 303) ^†^	2017 (*n* = 304) ^†^
*Demographic characteristics*			
% Men/CRC screening population	50.1 (49.2–50.8)	50.0 (49.2–50.8)	50.0 (49.3–50.8)
Age groups			
% 55–59/CRC screening population	25.9 (24.9–26.9)	25.6 (24.8–26.5)	30.0 (29.2–31.3)
% 60–64/CRC screening population	28.5 (27.8–29.5)	28.5 (27.8–29.2)	26.6 (25.9–29.2)
% 65–69/CRC screening population	25.8 (24.9–26.6)	25.1 (24.3–25.9)	25.1 (24.3–25.9)
% 70–74/CRC screening population	19.7 (18.8–20.6)	20.6 (19.7–21.8)	19.9 (19.0–21.0)
% With a partner	52.7 (51.1–53.9)	52.6 (51.1–53.8)	52.6 (50.9–53.7)
% Current non-Belgian/Dutch nationality	2.6 (1.90–4.20)	3.0 (2.1–4.6)	3.3 (2.3–4.8)
*Socioeconomic characteristics*			
Average income (per 1000 EUR) ^‡^	19.2 (18.1–20.6)	19.3 (18.1–20.9)	19.9 (18.7–21.4)
Provision level (Number, percentage)			
Level 1 (lowest)	59 (19.5%)	59 (19.5%)	60 (19.7%)
Level 2	65 (21.5%)	65 (21.5%)	65 (21.4%)
Level 3	81 (26.7%)	81 (26.7%)	81 (26.6%)
Level 4	53 (17.5%)	53 (17.5%)	53 (17.4%)
Level 5	18 (5.9%)	18 (5.9%)	18 (5.9%)
Level 6	14 (4.6%)	14 (4.6%)	14 (4.6%)
Level 7 (highest)	13 (4.3%)	13 (4.3%)	13 (4.3%)
Position in labour market			
% Wage earners	36.6 (34.8–37.9)	36.6 (34.5–37.9)	36.8 (34.8–38.1)
% Self-employed	7.9 (6.9–9.2)	8.0 (7.0–9.3)	8.1 (7.1–9.5)
% Jobseekers	1.8 (1.5–2.2)	1.8 (1.7–2.1)	1.6 (1.3–1.9)
% (Early)retired	19.7 (18.5–20.9)	19.9 (18.8–21.1)	20.1 (19.0–21.2)
% Higher education	44.4 (39.1–49.2)	44.8 (39.8–49.7)	45.5 (40.8–51.1)
*Health-related characteristics*			
‰ Disabled	6.4 (5.0–7.9)	6.5 (5.1–7.9)	6.4 (5.0–7.8)
% With at least 1 chronic disease	9.7 (8.8–10.6)	10.4 (9.6–10.5)	11.0 (10.1–12.1)
% With at least 1 GP visit in last 12 months	84.2 (82.1–86.0)	84.9 (82.7–86.6)	84.4 (82.3–86.4)
% With at least 2 preventive dental visits in 2 different years in last 3 years	34.7 (31.0–37.6)	37.4 (33.5–40.6)	40.1 (36.1–43.4)
% With a global medical dossier	74.8 (69.0–80.6)	78.4 (73.5–82.7)	82.0 (77.0–85.3)
Average number of patients per GP (per 100 patients) ^‡^	14.1 (12.1–16.2)	14.5 (12.6–16.8)	14.7 (12.5–17.4)

The percentages of men and age groups were captured for the colorectal cancer screening population in each municipality. Other characteristics were captured for the whole population in each municipality and were used as proxies for the colorectal cancer screening population. ^†^ Number of municipalities included in the analysis, for which data for all the study variables were available (cell ≥ 5 events). ^‡^ For statistical purposes, average income was divided by 1000 and average number of patients per GP was divided by 100 before inclusion into analyses. IQR, interquartile range; CRC, colorectal cancer.

**Table 3:** Univariable and multivariable associations between municipal characteristics and organised FOBT screening coverage.

	Univariable Analyses			Multivariable Analyses		
	OR	95% CI	*p* Value	OR	95% CI	*p* Value
*Demographic characteristics*						
Men/CRC screening population (%)	1.13	1.11–1.15	<0.001 *	1.13	1.11–1.14	<0.001 *
Age categories						
55–59/CRC screening population (%)	1.02	1.01–1.03	<0.001 *	1.005	0.995–1.014	0.37
60–64/CRC screening population (%)	0.986	0.973–0.9996	0.044 *	1.02	1.01–1.04	0.002 *
65–69/CRC screening population (%)	0.983	0.971–0.995	0.005 *	1.01	0.99–1.03	0.27
70–74/CRC screening population (%)	0.98	0.97–0.99	<0.001 *	0.98	0.97–0.99	<0.001 *
With a partner (%)	1.035	1.029–1.041	<0.001 *	1.035	1.029–1.040	<0.001 *
Current non-Belgian/Dutch nationality (%)	0.969	0.964–0.975	<0.001 *	0.962	0.957–0.967	<0.001 *
*Socioeconomic characteristics*						
Average income (per 1000 EUR) ^‡^	1.003	0.988–1.018	0.71	0.97	0.96–0.98	<0.001 *
Provision level						
Level 1 (lowest)	(ref)			(ref)		
Level 2	1.03	0.98–1.08	0.31	1.03	0.98–1.08	0.29
Level 3	1.02	0.97–1.07	0.54	1.02	0.97–1.06	0.52
Level 4	1.000	0.948–1.054	0.99	1.000	0.951–1.053	0.99
Level 5	0.99	0.91–1.08	0.83	0.99	0.92–1.07	0.83
Level 6	0.97	0.92–1.02	0.21	0.97	0.93–1.01	0.17
Level 7 (highest)	0.87	0.81–0.93	<0.001 *	0.87	0.82–0.92	<0.001 *
Position in labour market						
Wage earners (%)	1.02	1.01–1.03	<0.001 *	0.990	0.986–0.994	<0.001 *
Self-employed (%)	1.003	0.990–1.016	0.67	0.98	0.97–0.99	<0.001 *
Jobseekers (%)	0.92	0.90–0.95	<0.001 *	1.12	1.09–1.15	<0.001 *
(Early)retired (%)	1.004	0.994–1.014	0.42	0.977	0.971–0.983	<0.001 *
Higher education (%)	1.007	1.003–1.010	<0.001 *	1.010	1.008–1.011	<0.001 *
*Health-related characteristics*						
Disability (‰)	1.02	1.01–1.03	<0.001 *	1.024	1.015–1.034	<0.001 *
Chronic disease (%)	1.04	1.02–1.06	<0.001 *	0.991	0.978–1.004	0.18
GP visit (%)	1.043	1.040–1.047	<0.001 *	1.04	1.03–1.05	<0.001 *
Preventive dental visit (%)	1.017	1.014–1.021	<0.001 *	1.002	1.000–1.005	0.051
Global medical dossier (%)	1.019	1.017–1.021	<0.001 *	1.001	0.999–1.002	0.37
Patients per GP (per 100 patients) ^‡^	1.010	1.005–1.015	<0.001 *	1.009	1.005–1.014	<0.001 *

^‡^ For statistical purposes, average income was divided by 1000 and average number of patients per GP was divided by 100 before inclusion into analyses. * Statistically significant. FOBT, faecal occult blood test.

**Table 4:** Univariable and multivariable associations between municipal characteristics non-organised FOBT screening coverage.

	Univariable Analyses			Multivariable Analyses		
	OR	95% CI	*p* Value	OR	95% CI	*p* Value
*Demographic characteristics*						
Men/CRC screening population (%)	1.01	0.98–1.04	0.61	1.01	0.99–1.04	0.36
Age categories						
55–59/CRC screening population (%)	0.97	0.96–0.99	<0.001 *	1.016	0.999–1.034	0.07
60–64/CRC screening population (%)	1.07	1.05–1.10	<0.001 *	1.021	0.998–1.045	0.08
65–69/CRC screening population (%)	1.05	1.03–1.07	<0.001 *	0.98	0.95–1.01	0.14
70–74/CRC screening population (%)	0.97	0.96–0.99	0.002 *	0.981	0.964–0.999	0.037 *
With a partner (%)	1.002	0.992–1.012	0.68	1.002	0.994–1.011	0.64
Current non-Belgian/Dutch nationality (%)	0.997	0.989–1.005	0.51	0.998	0.990–1.007	0.71
*Socioeconomic characteristics*						
Average income (per 1000 EUR) ^‡^	1.001	0.985–1.016	0.95	1.03	1.01–1.06	0.010 *
Provision level						
Level 1 (lowest)	(ref)			(ref)		
Level 2	0.90	0.80–1.02	0.09	0.90	0.80–1.01	0.08
Level 3	0.95	0.85–1.06	0.38	0.95	0.85–1.06	0.35
Level 4	0.86	0.77–0.96	0.008 *	0.86	0.77–0.95	0.005 *
Level 5	0.94	0.82–1.07	0.35	0.94	0.82–1.06	0.31
Level 6	0.97	0.83–1.13	0.66	0.97	0.83–1.12	0.63
Level 7 (highest)	0.94	0.82–1.07	0.32	0.94	0.83–1.05	0.27
Position in labour market						
Wage earners (%)	1.002	0.993–1.010	0.74	0.987	0.977–0.996	0.005 *
Self-employed (%)	1.01	0.99–1.02	0.53	1.011	0.995–1.028	0.17
Jobseekers (%)	1.03	0.99–1.08	0.16	1.058	0.999–1.122	0.054
(Early)retired (%)	0.987	0.976–0.997	0.012 *	0.99	0.97–1.01	0.18
Higher education (%)	1.005	1.003–1.008	<0.001 *	1.001	0.998–1.004	0.53
*Health-related characteristics*						
Disability (‰)	0.9996	0.9821–1.0174	0.96	0.997	0.980–1.015	0.76
Chronic disease (%)	0.96	0.94–0.98	<0.001 *	0.98	0.95–1.02	0.35
GP visit (%)	1.011	1.001–1.021	0.038 *	1.03	1.02–1.04	<0.001 *
Preventive dental visit (%)	0.997	0.992–1.003	0.32	0.998	0.991–1.005	0.55
Global medical dossier (%)	1.004	1.000–1.008	0.033 *	1.025	1.018–1.031	<0.001 *
Patients per GP (per 100 patients) ^‡^	1.018	1.013–1.024	<0.001 *	1.021	1.016–1.026	<0.001 *

^‡^ For statistical purposes, average income was divided by 1000 and average number of patients per GP was divided by 100 before inclusion into analyses. * Statistically significant FOBT, faecal occult blood test.

## Data Availability

Data on organised and non-organised screening coverages, gender and age-specific proportions of the target screening population can be requested by contacting the Centre for Cancer Detection in Flanders at https://www.bevolkingsonderzoek.be/ (accessed on 6 August 2021). Data on the other demographic, socioeconomic and health-related variables are publicly available on the https://provincies.incijfers.be/databank (accessed on 6 August 2021) website.

## Supplementary Materials

The following are available online at https://www.mdpi.com/article/10.3390/ijerph18168373/s1, Figure S1: Causal directed acyclic graphs (DAGs) constructed to identify covariates for adjustment in multivariable analyses. After covariate adjustment, no causal paths (indicated by purple lines) are present.
