# Supplementary material for: Population-Based Data Reveal Factors Associated with Organised and Non-Organised Colorectal Cancer Screening: An Important Step towards Improving Coverage
Source: Int J Environ Res Public Health. 2021 Aug 7;18(16):8373. doi: 10.3390/ijerph18168373 (PMC8392464; doi:10.3390/ijerph18168373)

Supplemental material for

Population-based data reveal factors associated with organised and non-organised colorectal cancer screening: an important step towards improving coverage

Supplementary figure

Supplementary Figure S1 Causal directed acyclic graphs (DAGs) constructed to identify covariates for adjustment in multivariable analyses. After covariate adjustment, no causal paths (indicated by purple lines) are present.

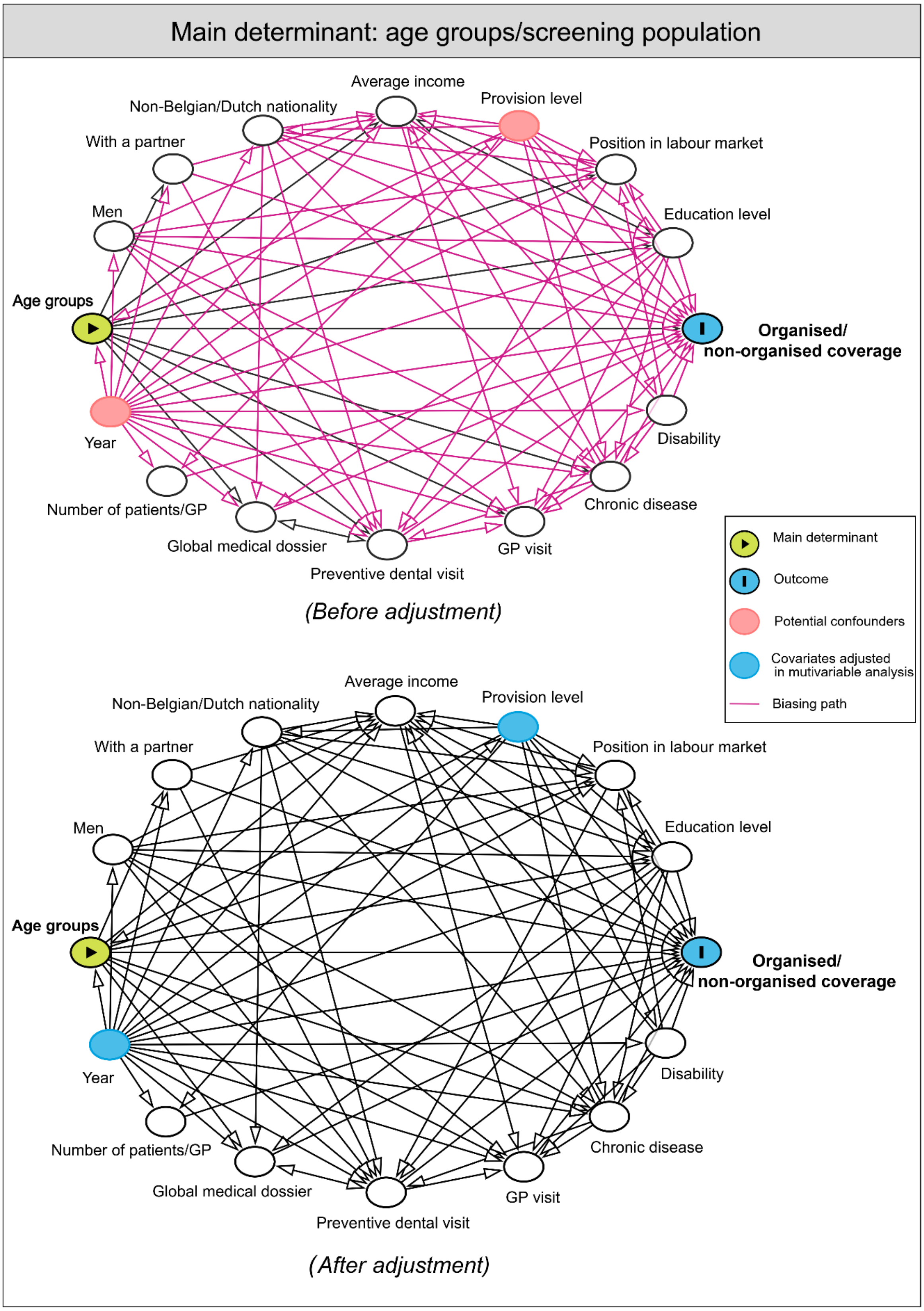

Supplementary Figure S1 Causal directed acyclic graphs (DAGs) constructed to identify covariates for adjustment in multivariable analyses. After covariate adjustment, no causal paths (indicated by purple lines) are present.

Supplementary Figure S1 Continued.

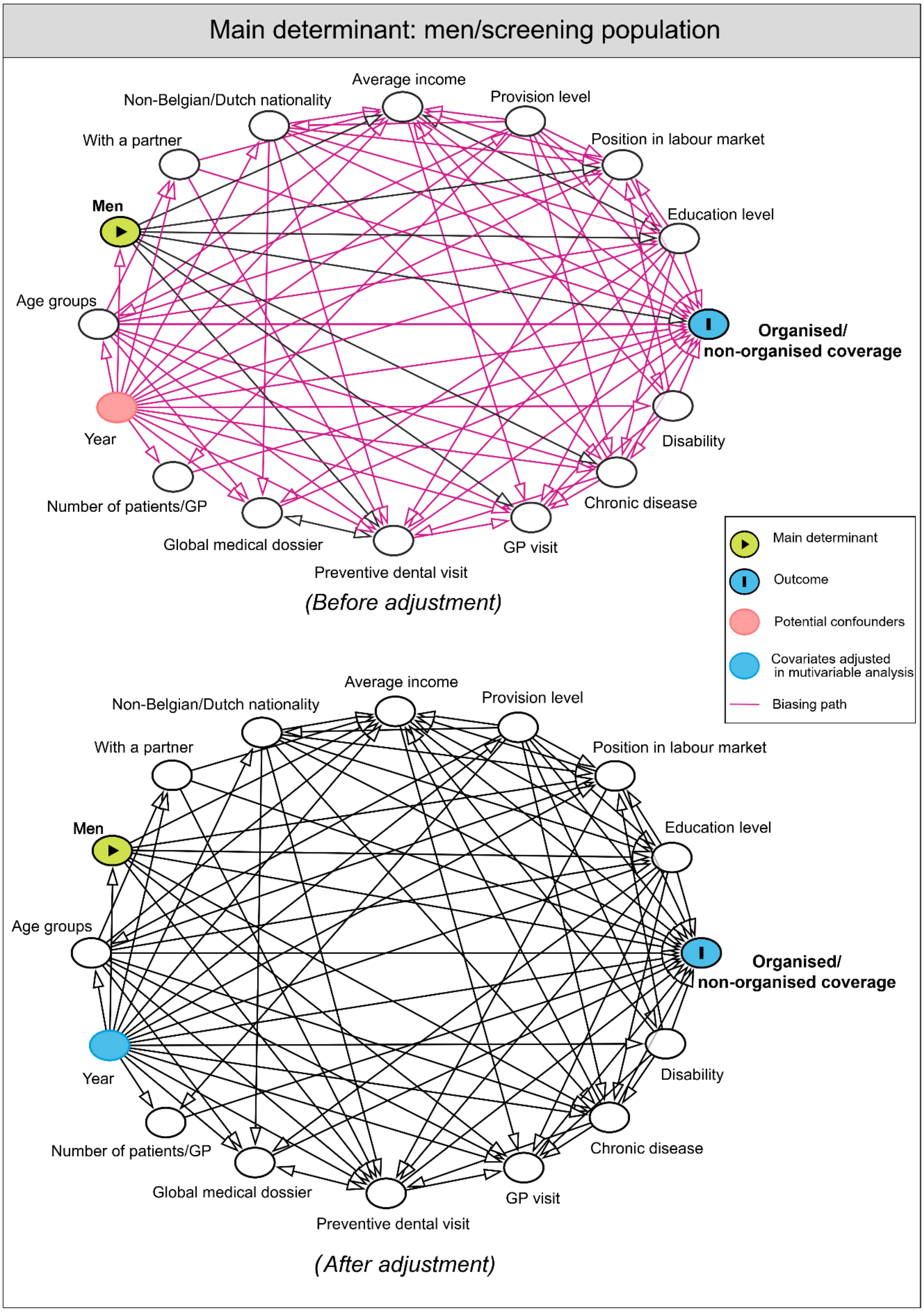

Supplementary Figure S1 Continued.

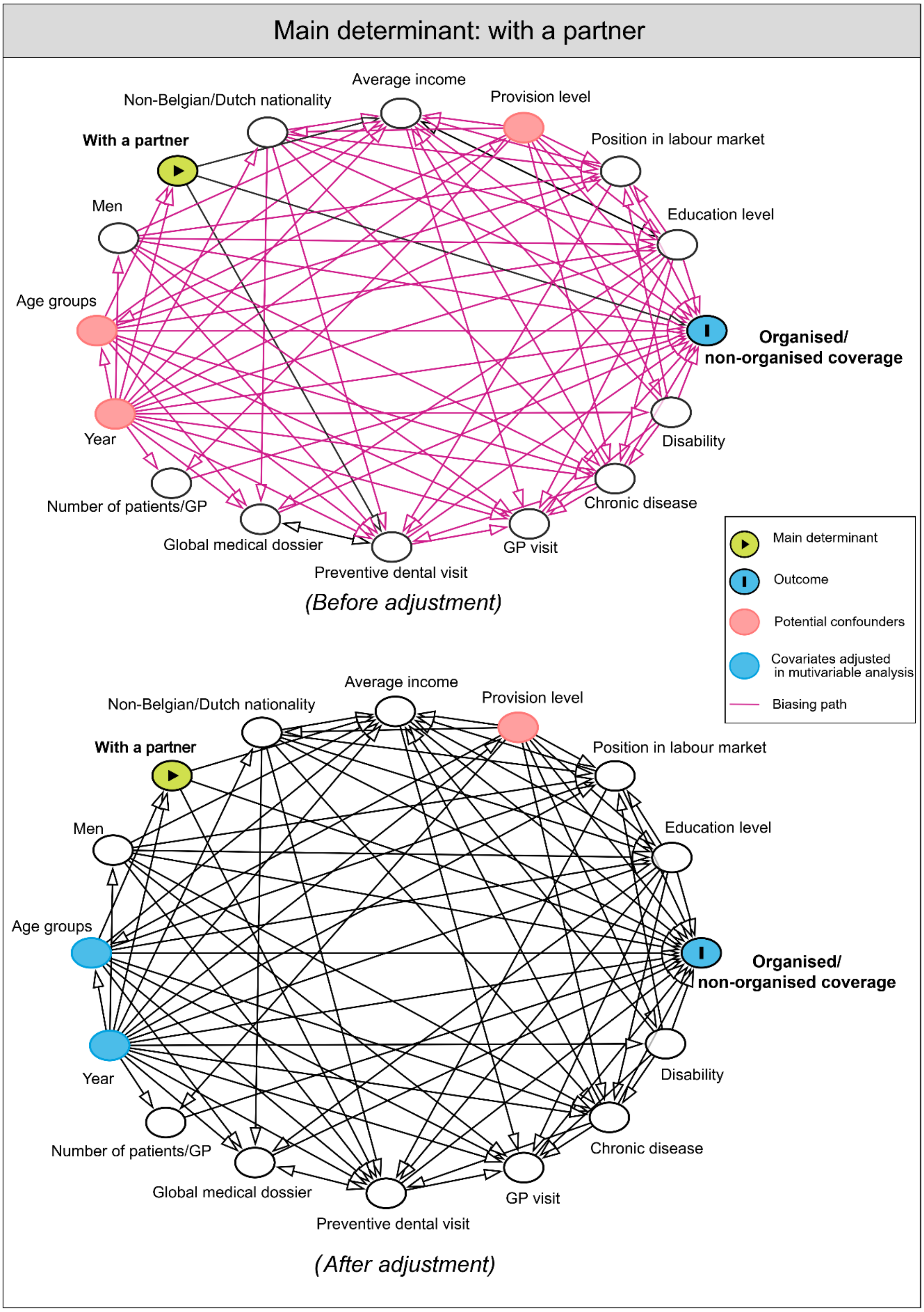

Supplementary Figure S1 Continued.

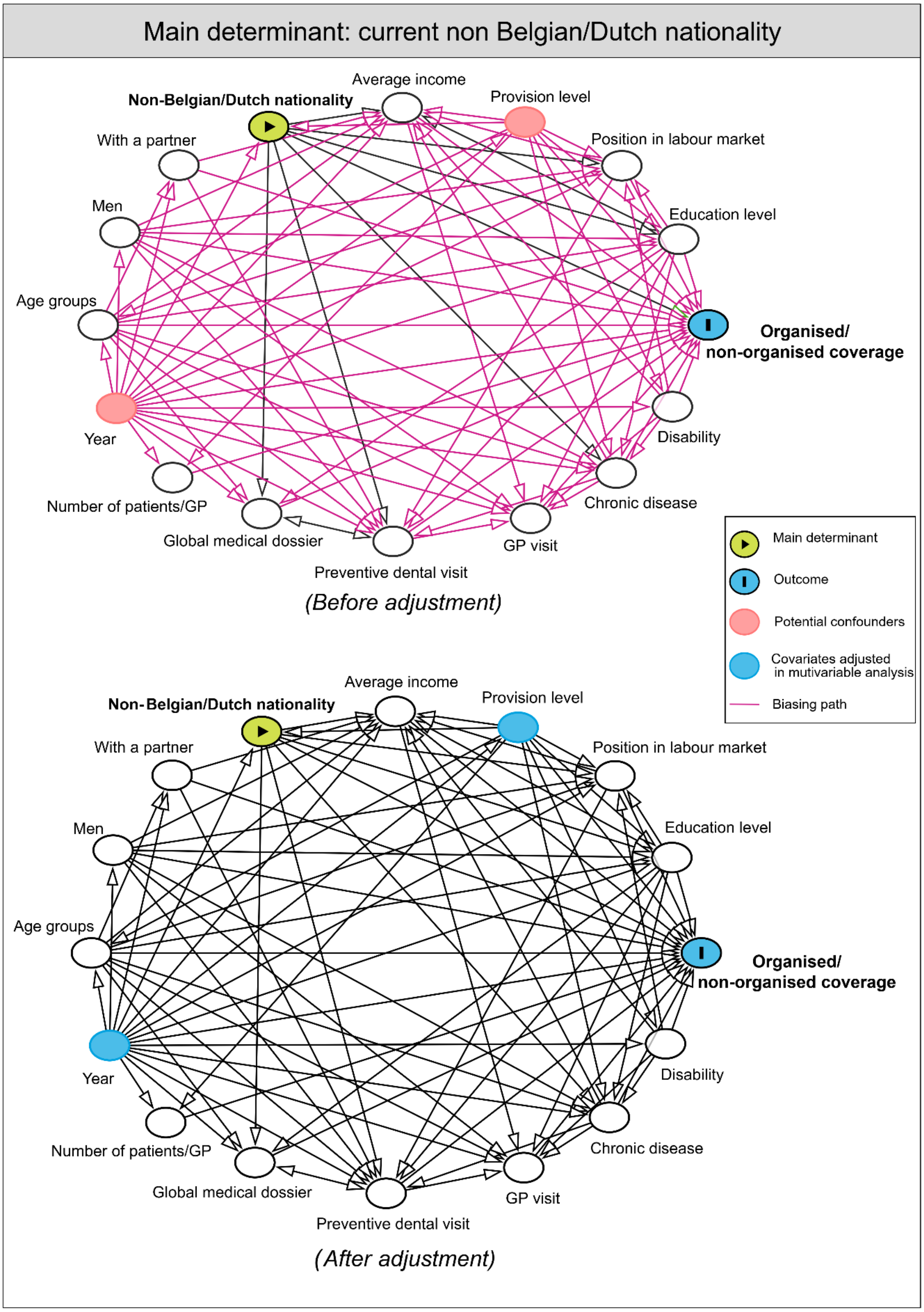

Supplementary Figure S1 Continued.

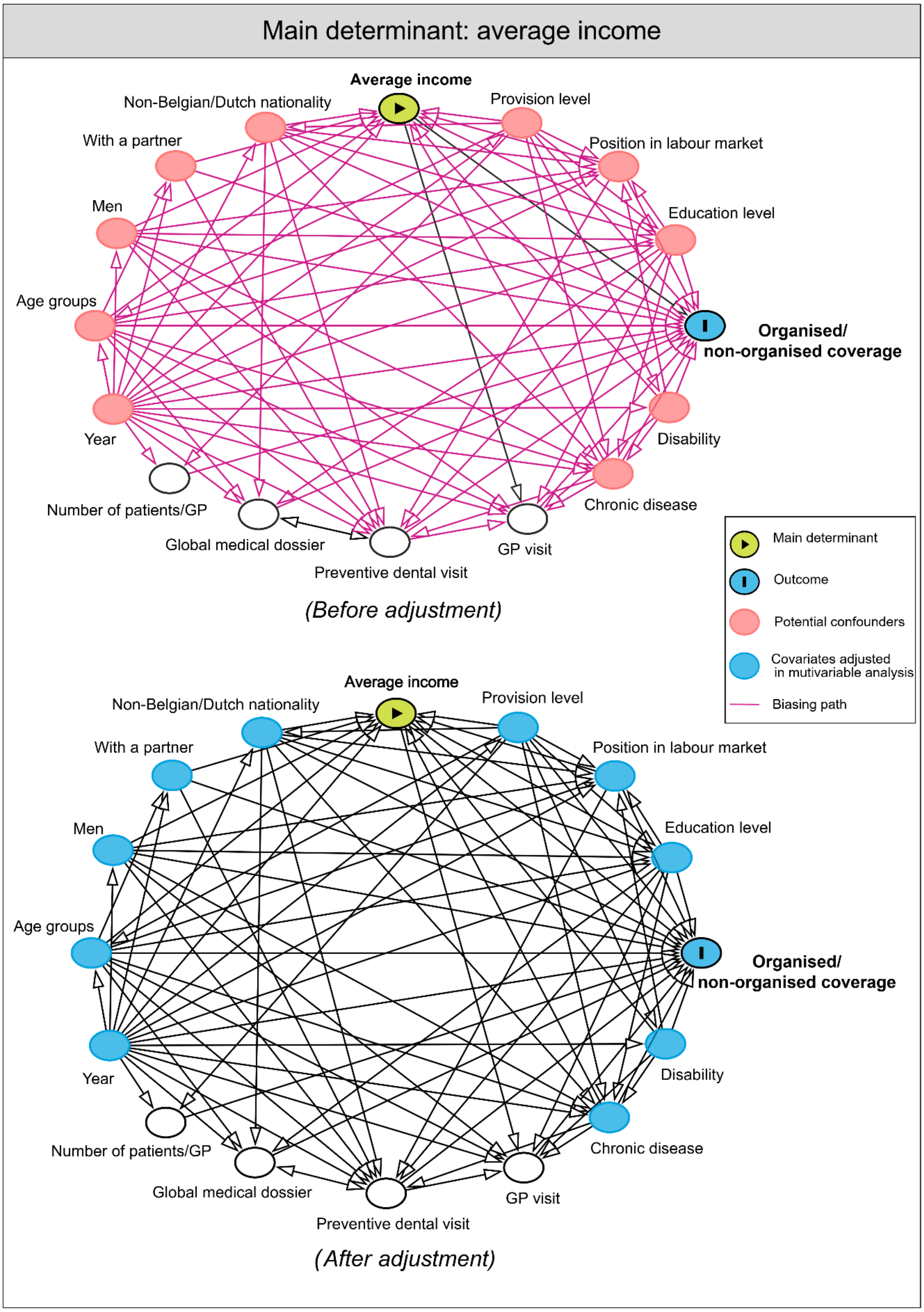

Supplementary Figure S1 Continued.

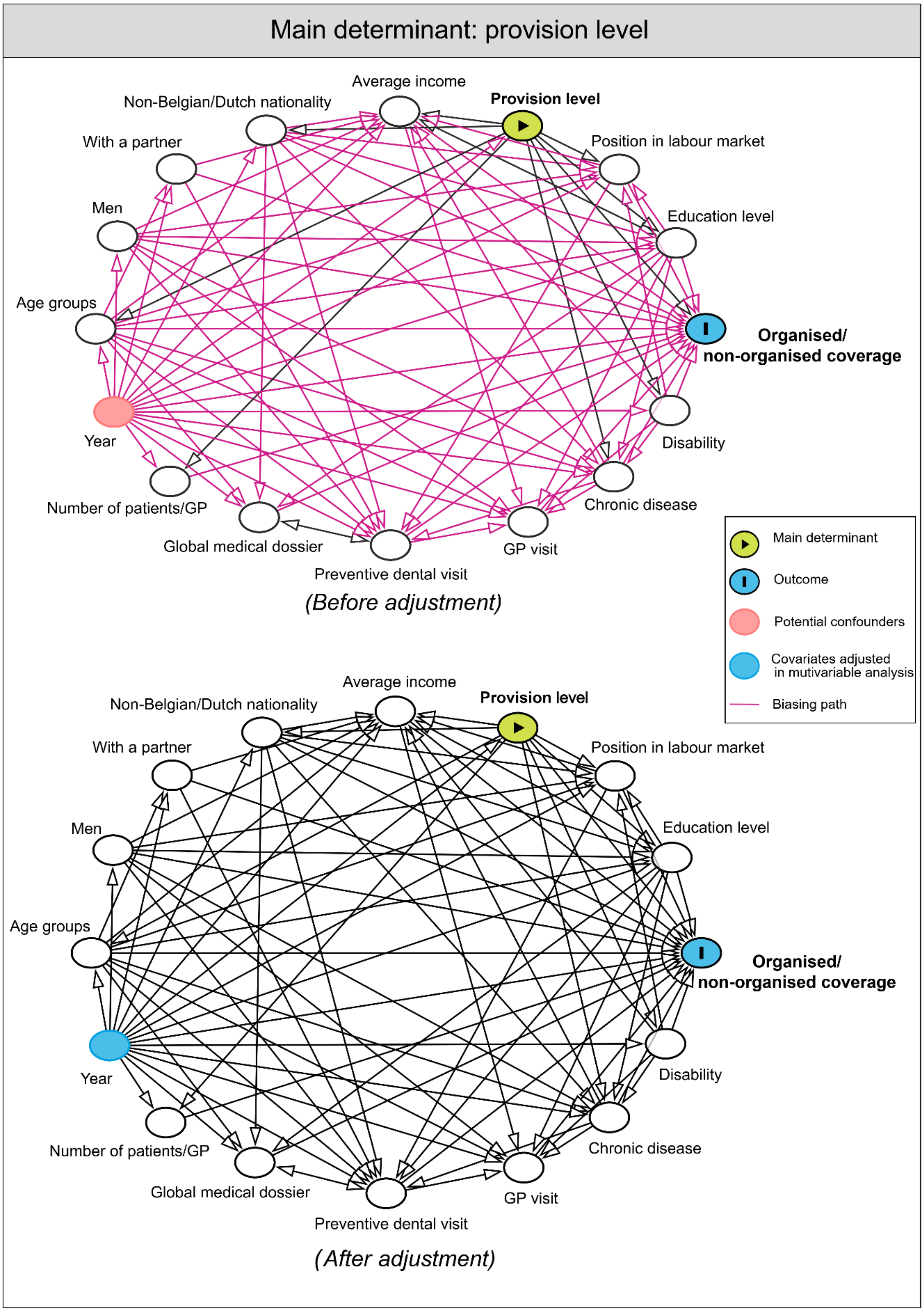

Supplementary Figure S1 Continued.

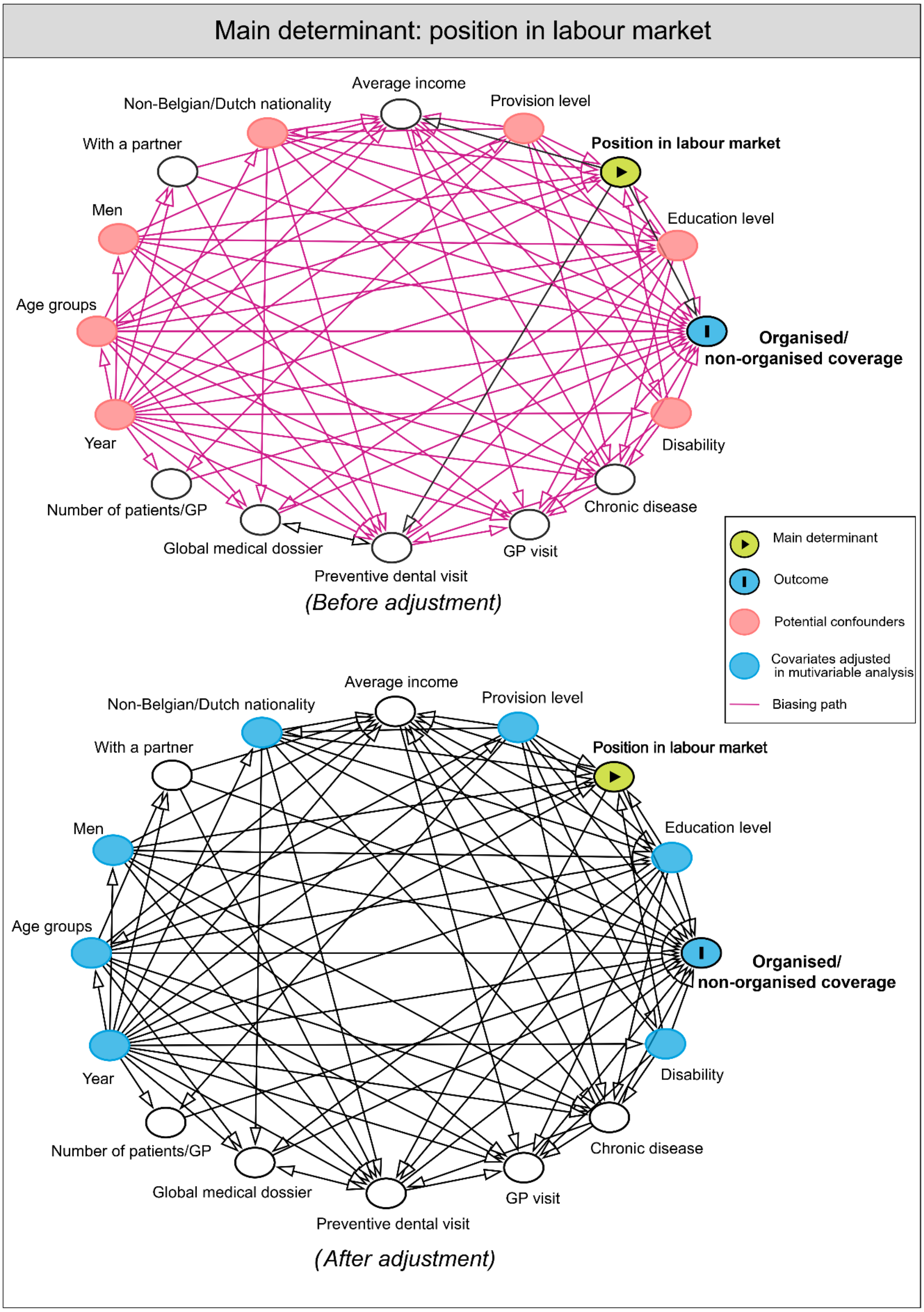

Supplementary Figure S1 Continued.

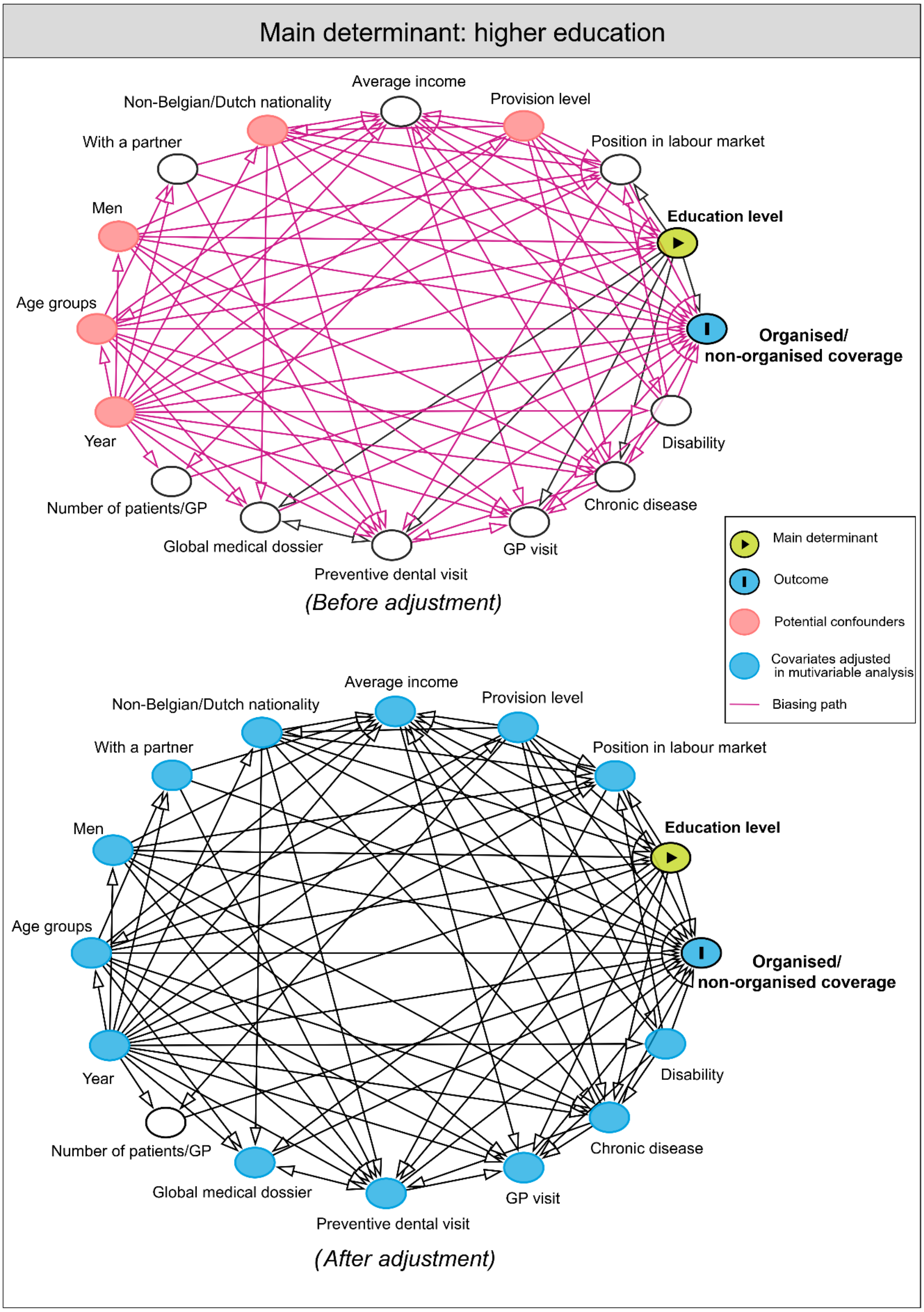

Supplementary Figure S1 Continued.

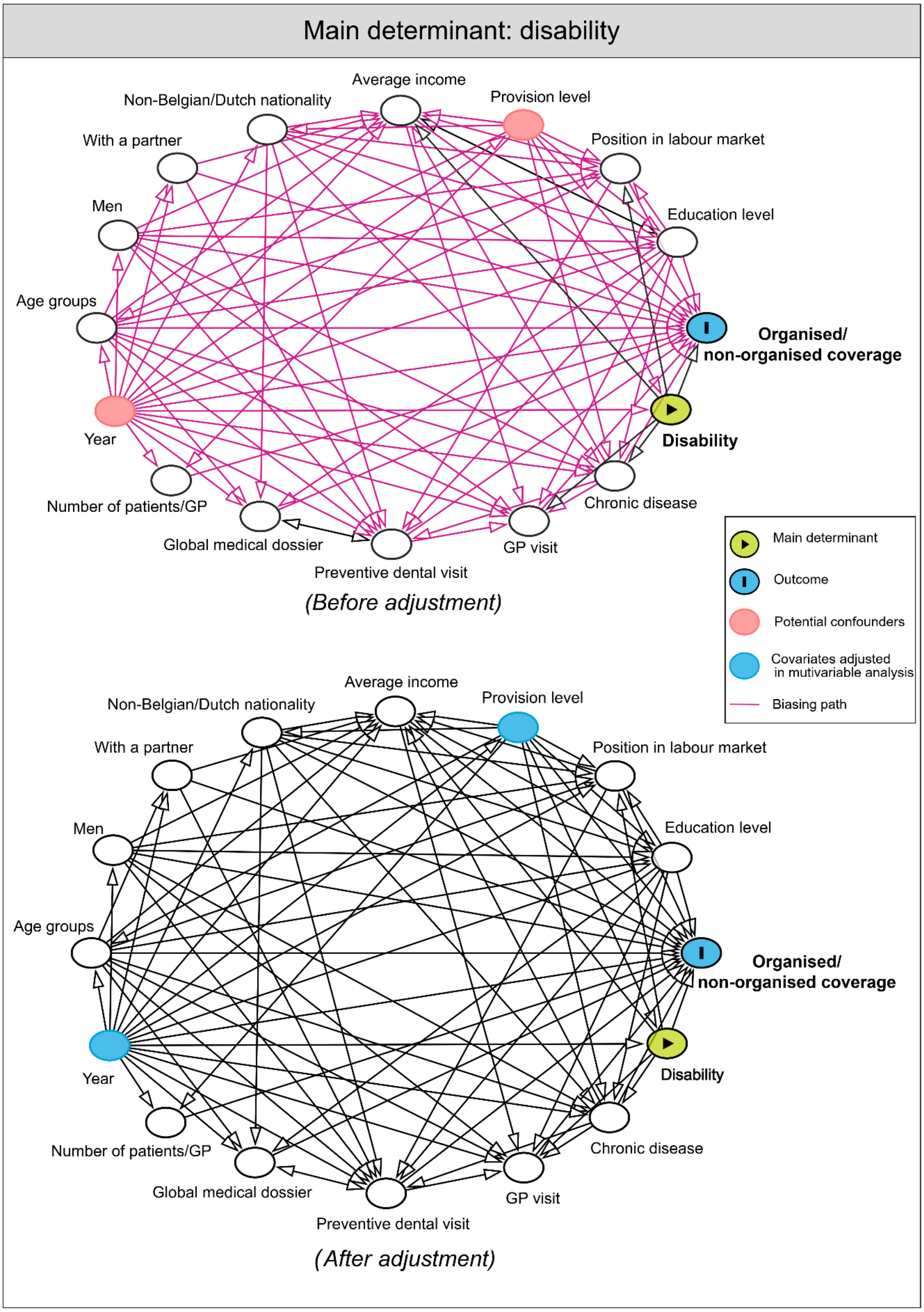

Supplementary Figure S1 Continued.

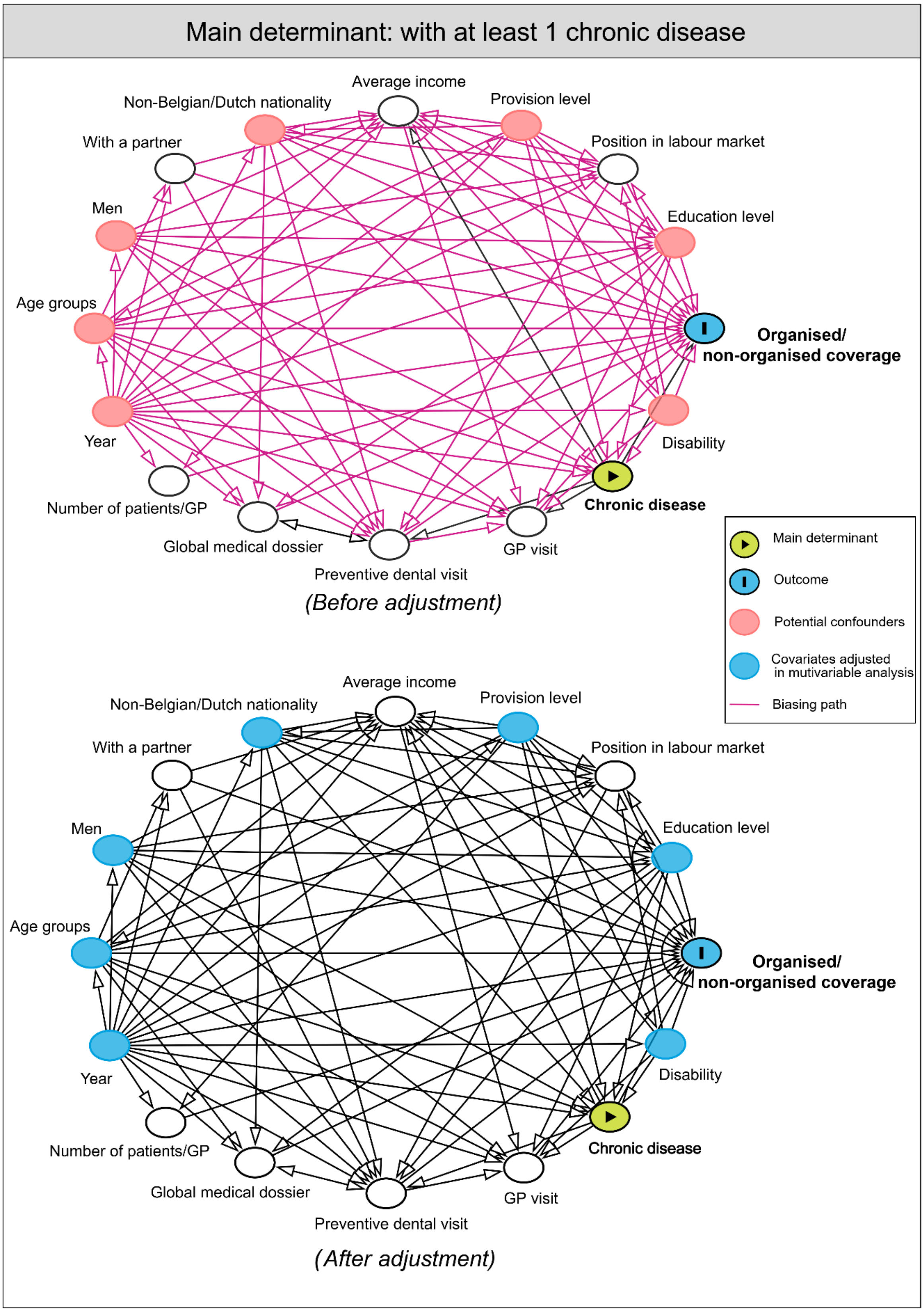

Supplementary Figure S1 Continued.

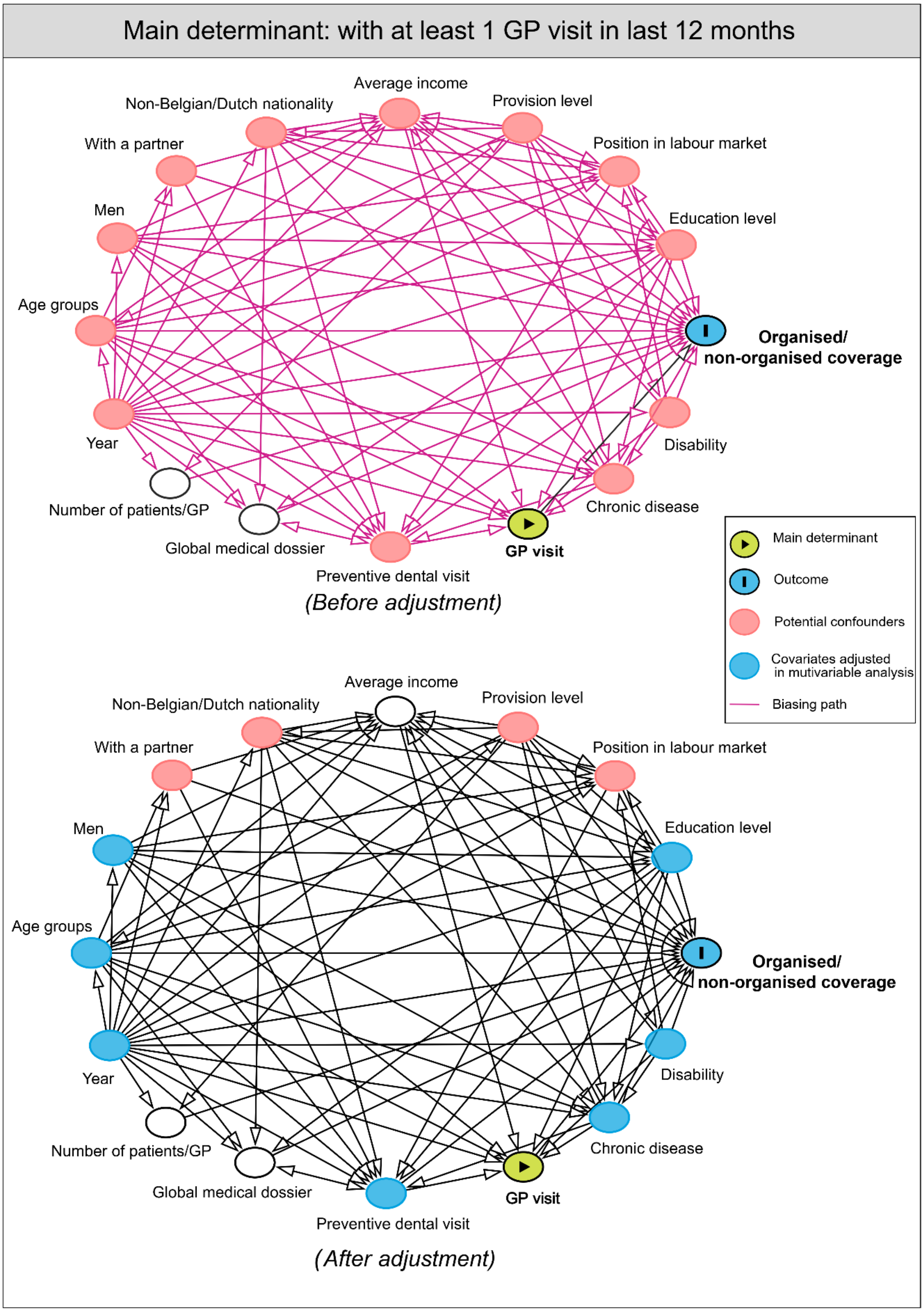

Supplementary Figure S1 Continued.

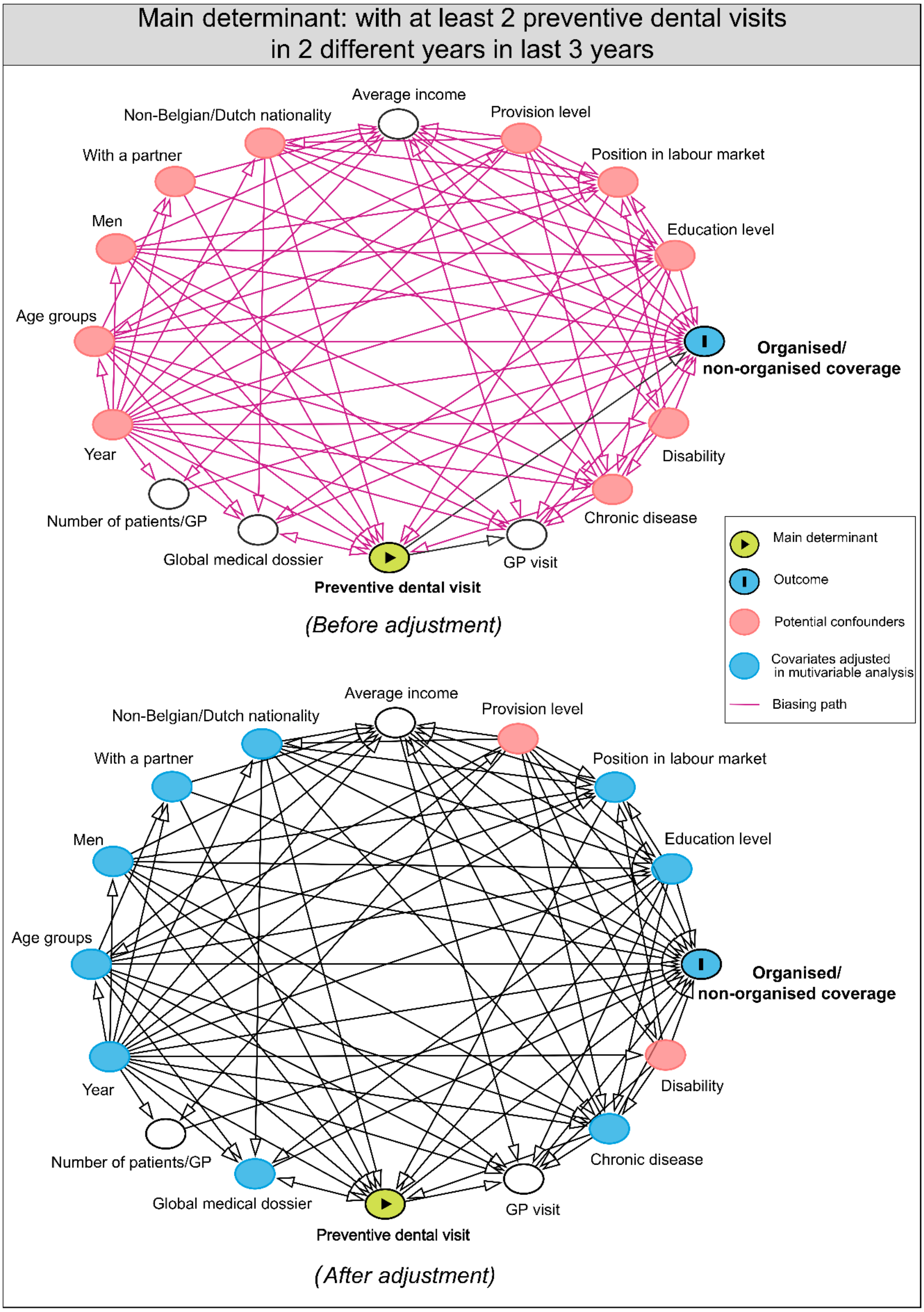

Supplementary Figure S1 Continued.

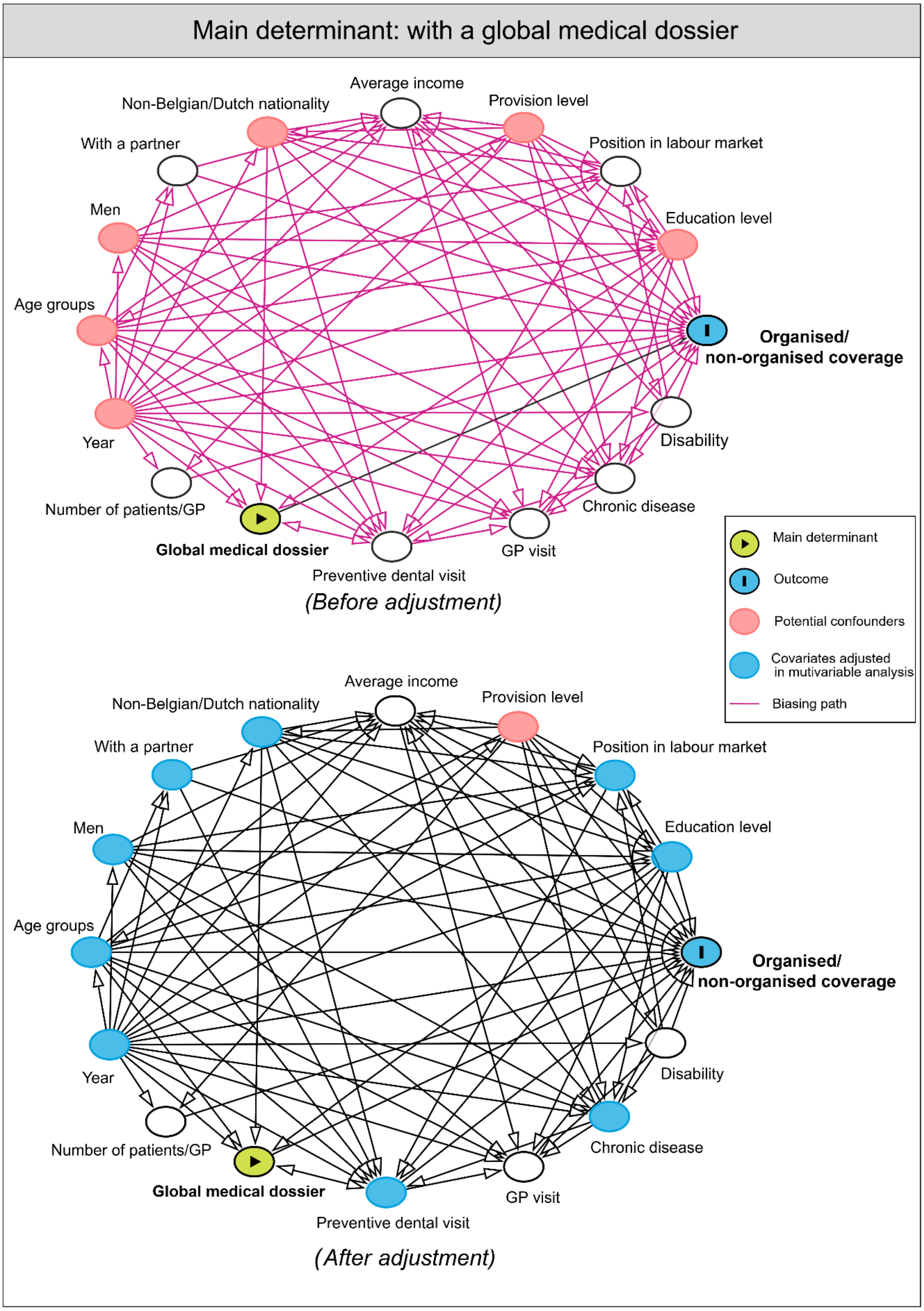

Supplementary Figure S1 Continued.

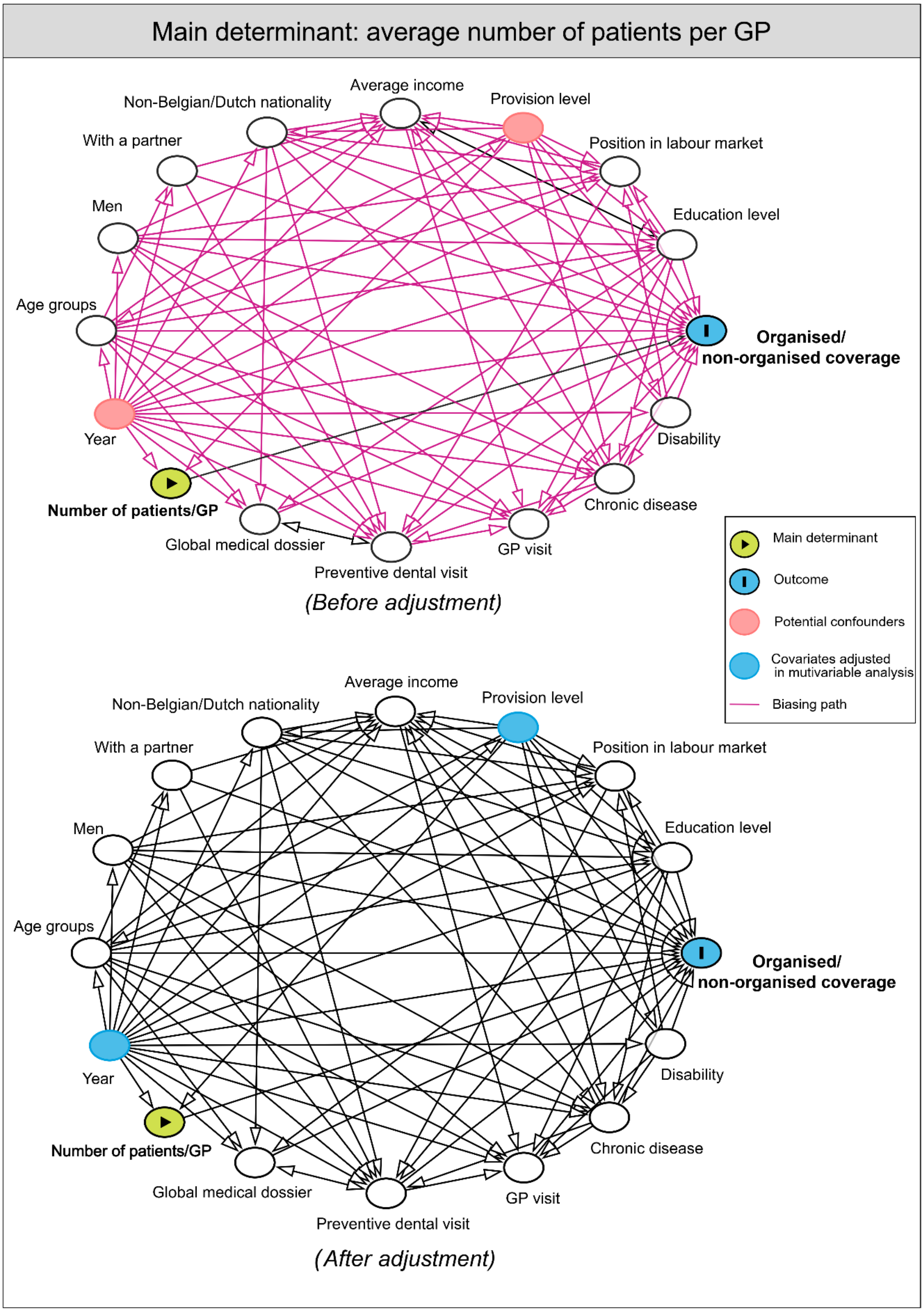

Supplement: Supplementary file 1 [file ijerph-18-08373-s001.zip › ijerph-1291093-supplementary.pdf]
